# Long-Term Neurological Outcomes in West Nile Virus–Infected Patients: An Observational Study

**DOI:** 10.4269/ajtmh.14-0616

**Published:** 2015-05-06

**Authors:** Jill E. Weatherhead, Vicki E. Miller, Melissa N. Garcia, Rodrigo Hasbun, Lucrecia Salazar, Mazen M. Dimachkie, Kristy O. Murray

**Affiliations:** Baylor College of Medicine, Department of Pediatrics, Houston, Texas; The University of Texas Health Science Center at Houston, Houston, Texas; The University of Kansas Medical Center, Kansas City, Kansas

## Abstract

The Houston West Nile Cohort (HWNC) was founded in 2002 when West Nile virus (WNV) reached Houston, TX. The long-term outcomes following WNV infection are still mostly unknown, though neurological abnormalities up to 1 year postinfection have been documented. We report an observational study of neurological abnormalities at 1–3 and 8–11 years following WNV infection in the HWNC. We conducted standard neurological examinations at two separate time points to assess changes in neurological status over time. The majority of patients (86%, 30/35) with encephalitis had abnormal neurological exam findings at the time of the first assessment compared with uncomplicated fever (27%, 3/11) and meningitis (36%, 5/14) cases. At the time of the second assessment, 57% (4/7) of West Nile fever (WNF), 33% (2/6) of West Nile meningitis (WNM), and 36% (5/14) of West Nile encephalitis (WNE) had developed new neurological complications. The most common abnormalities noted were tandem gait, hearing loss, abnormal reflexes, and muscle weakness. Long-term neurological abnormalities were most commonly found in patients who experienced primary WNV encephalitis. New abnormalities may develop over time regardless of initial clinical infection. Future studies should aim to differentiate neurological consequences due to WNV neuroinvasive infection versus neurological decline related to comorbid conditions.

## Introduction

The first outbreak of West Nile virus (WNV) in the Western Hemisphere occurred in New York City in 1999.^1^ Subsequently, the virus spread rapidly south and westward throughout the United States reaching Harris County, Texas in 2002. By the end of 2004, human infection had been documented in all states except Washington, Hawaii, and Alaska.^2^,^3^ Between 1999 and 2013, more than 39,000 cases of clinical WNV were reported to Centers for Disease Control and Prevention (CDC).^4^

WNV infection is most commonly asymptomatic with approximately 20% infected persons found to have clinically apparent disease.^5^–^7^ The majority of people with symptoms present with fatigue, fever, and headache and less commonly with myalgia, muscle weakness, rash, difficulty concentrating, neck pain, arthralgia, vomiting, diarrhea, and sensitivity to light.^8^,^9^ West Nile neuroinvasive disease (WNND), characterized as meningitis, encephalitis, and/or acute flaccid paralysis, occurs in as few as 1 in 150 infected persons.^6^ WNV meningitis is defined clinically as fever, pleocytosis in the cerebrospinal fluid (CSF), and clinical evidence of meningeal inflammation (nuchal rigidity, photophobia, and nausea/vomiting). WNV encephalitis further requires the presence of prolonged altered mental status (> 24 hours), seizures, or focal neurological abnormalities.^10^,^11^ Among the patients who meet clinical criteria for WNND, acute case fatality is approximately 10%.^1^ Patients presenting with clinically compatible symptoms are typically diagnosed with WNV infection by detection of anti-WNV IgM using monoclonal antibody capture enzyme-linked immunosorbent assay (MAC-ELISA) in the serum or CSF.^5^,^12^

Although short-term follow up studies have shown persistent neurologic deficits in patients with WNND, little is known about the long-term neurologic sequelae associated with West Nile fever (WNF), meningitis (WNM), and encephalitis (WNE).^13^ In this study, we longitudinally examined a large cohort of patients with a history of WNV infection to determine long-term neurologic outcomes at 1–3 and 8–11 years post–acute WNV infection.

## Methods

Beginning in 2002, Houston metropolitan area residents with suspected WNV infection who tested positive for anti-WNV IgM antibodies by ELISA were reported to local public health officials. Depending on clinical presentation, cases were classified as follows: encephalitis/meningoencephalitis/encephalomyelitis (WNE) diagnosed by positive anti-WNV IgM antibody, CSF pleocytosis, and global cerebral dysfunction with altered mental status with or without focal neurological signs and symptoms or seizures; WNM diagnosed by positive anti-WNV IgM antibody, CSF pleocytosis, and meningeal signs and symptoms including headache and neck stiffness, and non-neuroinvasive viral syndrome with fever diagnosed by positive anti-WNV IgM antibody; and WNF with no neurological abnormalities identified. Cases identified through public health surveillance were invited to participate in the Houston West Nile Cohort (HWNC). Those available and interested were consented prior to study enrollment. The primary objectives of the longitudinal cohort study were to identify risk factors for WNND and to follow long-term clinical outcomes. No one was denied participation based on age, gender, race, or ethnicity. This study was reviewed and approved by the Committee for the Protection of Human Subjects at the University of Texas Health Science Center at Houston (HSC-SPH-03-039) and Baylor College of Medicine's IRB (H-30533). As a component of this study, cohort participants were invited to have a complete neurological examination at two different time points following infection, with the first assessment occurring between 2005 and 2006 (1–3 years postinfection) and the second assessment occurring between 2012 and 2013 (8–11 years postinfection).

All neurological examinations were performed by physicians. The examination included assessment of level of consciousness, cranial nerve, cerebellar function, motor and sensory functions, reflexes, and gait. Data from the neurological exams were recorded on the Neurological Examination Form at the time of the exam. A descriptive analysis of abnormal neurological outcomes by clinical presentation (WNF, WNM, and WNE) was performed. Univariate analysis was performed to examine the relationship between age (55 years and older), WNE, and gender with the outcome of an abnormal neurological exam. All variables with *P* < 0.25 on univariate analysis were entered into a multivariate logistic regression model to determine which variables were independently, statistically (*P* ≤ 0.05) associated with having an abnormal neurological exam.

## Results

### Primary neurological assessment (1–3 years postinfection).

**Figure 1.**
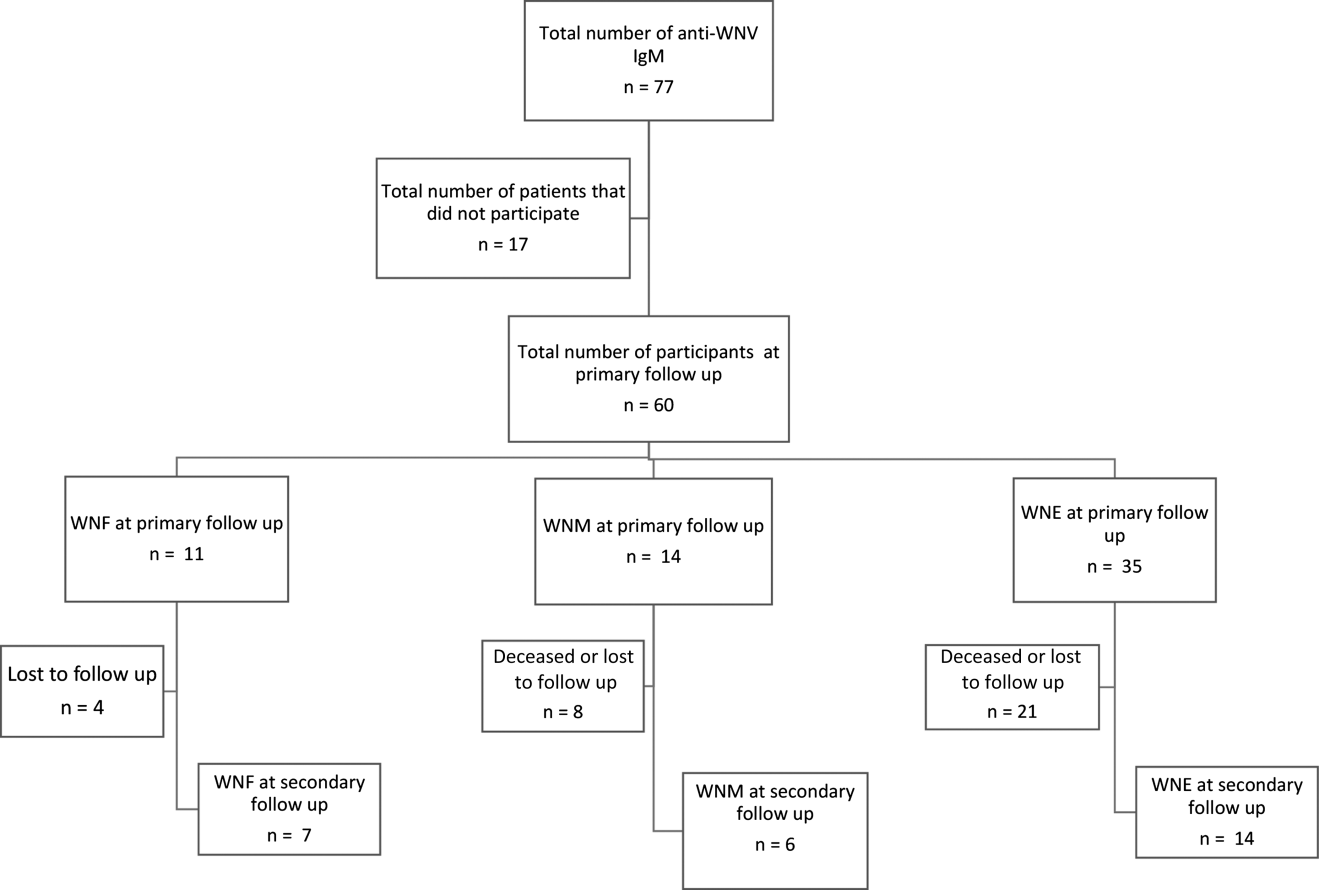
Flow chart of West Nile virus–positive participants in Houston enrolled between 2002 and 2004.

Sixty of the initial 77 (78%) cohort participants who were infected between 2002 and 2004 agreed to participate and were available for primary neurological assessment. Of the 60 participants, 11 had an initial clinical presentation of WNF, 14 had WNM, and 35 had WNE (Figure 1).

The demographics of the 60 patients are included in Table 1. The median age in years was older in the WNE compared with WNM and WNF groups (68 years versus 46 and 54 years, respectively). Furthermore, the population with WNE had a higher prevalence of comorbidities, particularly hypertension (65%), diabetes mellitus (31%), and history of stroke (22%). The majority of patients with WNM and WNE were male.

The primary neurologic exams of the 11 patients with WNF at 1–3 years postinfection were normal with exception of three (27%) patients (Table 2). One patient was found to have abnormal hearing prior to acute infection. The second patient had ataxia with tandem walking and decreased hearing bilaterally, although onset of hearing loss after acute infection was not documented. The third patient was found to have decreased right arm strength; abnormal tandem gait, toe, and heel walking; and positive Romberg sign. Cranial nerves, muscle strength, sensation, cerebellar examination, and reflexes were normal in all patients.

The primary neurologic exams among the 14 patients with WNM were also largely unremarkable (Table 2). Cranial nerve examinations were all normal. Sensory examination was normal in all participants with the exception of two patients. One patient with a history of diabetes mellitus and hypertension had decreased pinprick sensation in both feet and decreased vibratory sensation in the patient's right foot, consistent with peripheral neuropathy. The second patient with a history of stroke had decreased light touch in bilateral feet and left shin, decreased vibratory sensation in left foot, and intermittent left facial numbness. This patient also had abnormal toe walking because of left foot plantar flexion weakness. All other patients had normal strength throughout. All participants had normal motor strength, absence of clonus, and normal Babinski reflexes. One patient had symmetric but decreased reflexes in the lower extremities. All had normal gait and were able to perform the heel walking test. Two patients had abnormal tandem gait; however, both had significant comorbidities. One participant with abnormal tandem gait had a history of prior alcohol and drug abuse and the other had a history of diabetes mellitus. Coordination was also unremarkable with normal rapid alternating movements, heel/shin, and finger-to-nose tests. One patient had a slight tremor in the left hand.

A total of 35 patients with WNE were evaluated at primary follow-up, and 30 (86%) were found to have abnormal neurological exams (Table 2). Thirteen patients (37%) had abnormal motor strength and 9 patients (26%) had abnormal reflexes, although some reported strength and reflex abnormalities prior to WNV infection. Eleven patients (31%) had abnormal vibratory sensation. One patient with unilateral decreased vibratory sensation also had ipsilateral tongue deviation. Seven patients had tremors (20%), two of which were intention tremors. Hearing abnormalities were found in 16 (46%) patients with WNE, with 5 of these 16 patients (31%) reporting some hearing loss prior to infection. One patient who presented with decreased visual acuity at the onset of WNV continued to have abnormal visual acuity 3 years postinfection. Additional cranial nerve abnormalities were noted: lateral gaze palsy with nystagmus (one patient), unilaterally trapezius muscle weakness (one patient), and tongue deviation without prior history of stroke (one patient). Most notably, tandem gait abnormalities occurred in 60% WNE patients, and many of these patients had abnormal Romberg, gait, and/or toe and heel walking. Two patients had abnormal heel-to-shin exam but none of the patients had abnormal rapid alternating movements or abnormal finger-to-nose testing.

### Secondary neurological assessment (8–11 years postinfection).

A total of 27 patients (45%) were available for the second neurological assessment (8–11 years post–WNV infection). Of the 33 patients who were unavailable, 12 (36%) patients had died before the second neurological exam, 9 (27%) had moved or were lost to follow-up, 7 (21%) could not make it to the clinic for the exam, and 4 (12%) refused further participation.

A total of 7 patients from the original 11 patients (63% retention) with WNF had a neurologic exam at second follow up (Supplemental Table 1). One patient who had documented abnormal tandem gait and abnormal hearing following WNF at primary follow-up was found to have severe neurological sequelae on second exam. She was nonambulatory, and required complete assistance for activities of daily living. She was nonverbal, aphasic, and unresponsive to commands. She had severe contractures of all extremities with increased tone. A second patient with no history of comorbidities and a normal neurologic exam at the time of the first neurological assessment was found to have new bilateral lower extremity decreased sensation to pinprick. A third patient, with a history of hepatitis B virus infection, who previously was found to have decreased right arm strength and abnormal tandem gait, Romberg, toe walking, and heel walking, had improved arm strength. However, the patient continued to have difficulty with tandem gait, Romberg, toe walking, heel walking, and developed abnormal pinprick sensation in upper extremities. The fourth patient who was otherwise healthy and had a normal primary neurologic exam was subsequently found to have abnormal pinprick sensation, left hand weakness, and abnormal tandem gait on secondary assessment. The remaining three patients had normal primary and secondary assessments.

A total of 6 patients from the original 14 patients (43% retention) with WNM were evaluated at the secondary neurological assessment (Supplemental Table 1). Two patients had normal exams on primary and secondary neurologic exams. Two patients at secondary follow-up exam originally presented with abnormal tandem gait. The first patient, who had a history of chronic alcohol use and received treatment of hepatitis C infection, was found to have continued difficulty with tandem gait. The patient also had bilateral lower extremity weakness, decreased symmetric reflexes, and abnormal pinprick sensation in bilateral lower extremities. The second patient had a history of diabetes mellitus and hypertension and also continued to have abnormal tandem gait on follow-up exam. This patient was also found to have abnormal sensation to pinprick and vibrator on primary exam, and experienced continued abnormal sensation at the time of the follow-up. We cannot rule out the influence of diabetic neuropathy on this patient. One patient who had decreased symmetric reflex present on primary follow-up saw resolution in abnormal reflexes on secondary follow-up. A fourth patient with no past medical history had new abnormal pinprick sensation in bilateral lower extremities.

A total of 14 patients of the original 35 patients with WNE (40% retention) were evaluated at secondary follow-up (Supplemental Table 2); 11 (31%) patients had died before the time of the second exam (Supplemental Table 1). Out of the 14 WNE patients who had a second exam, six had abnormal tandem walking and six had abnormal hearing exam at the primary follow-up neurologic exam. Of the six patients who had abnormal tandem gait at primary examination, all continued to have abnormal tandem gait. Of the six patients who had abnormal hearing, all continued to have hearing difficulties. Four patients who had abnormal reflexes documented at primary neurologic follow-up were evaluated at secondary follow-up, and had persistent reflex abnormalities. Two patients who had previously had normal reflexes developed abnormal reflexes on secondary exam. The first patient had a history of a stroke, and developed decreased reflexes in upper extremities on secondary exam. The second patient had a history of hypertension and developed decreased symmetric reflexes in upper and lower extremities. Three of the 14 patients had documented muscle weakness during the primary neurologic exam, and two of these patients continued to have significant muscle weakness on secondary follow-up. One patient with a history of hypertension developed symmetric decreased strength in upper and lower extremities on secondary follow-up. Of the two patients who previously had a tremor, one patient experienced resolution of the tremor and one patient had a continued tremor on the second exam. Furthermore, one patient developed a new tremor that had previously been absent.

**Figure 2.**
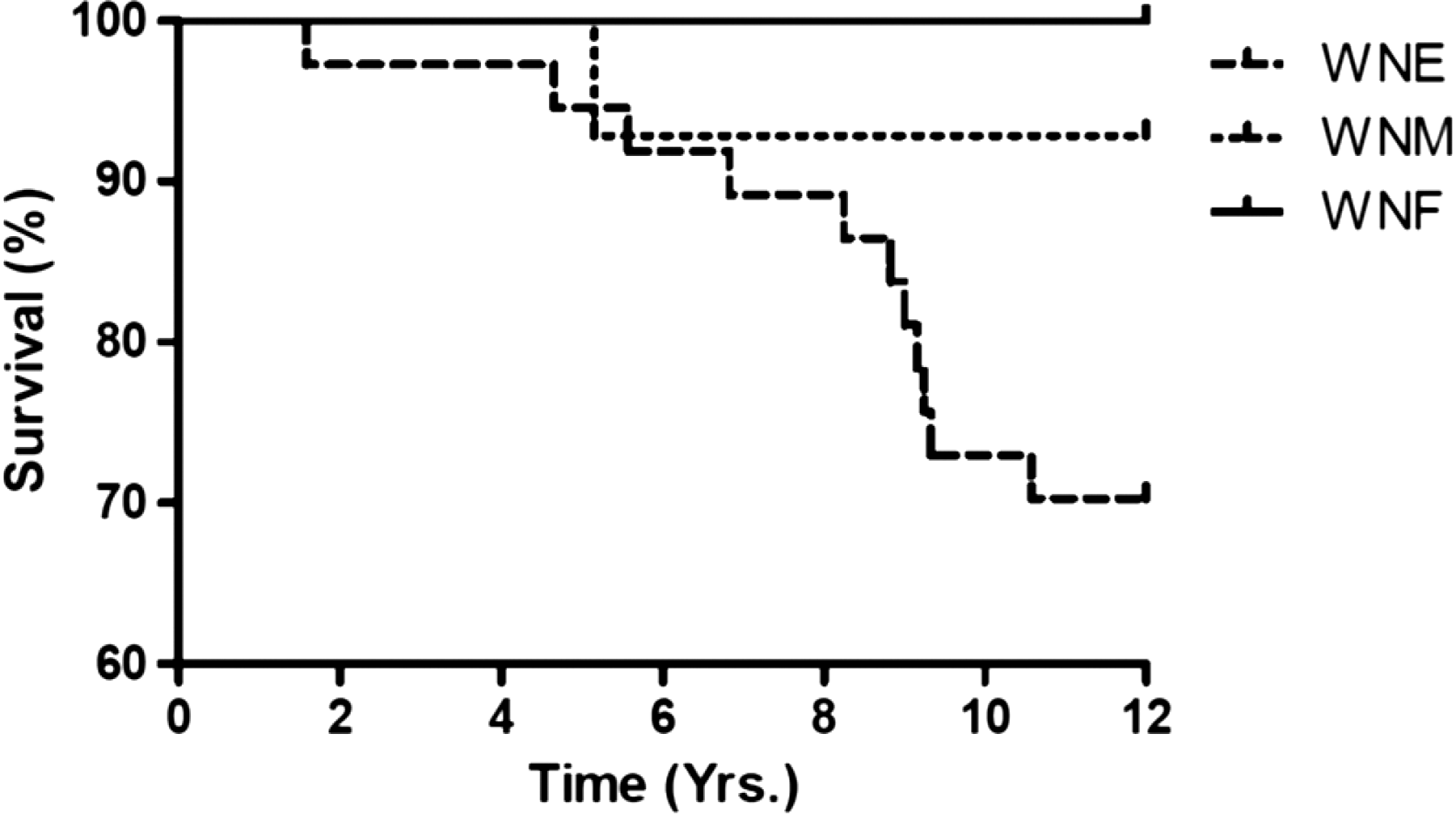
Kaplan Meier curve of deaths among all acute clinical presentations.

**Figure 3.**
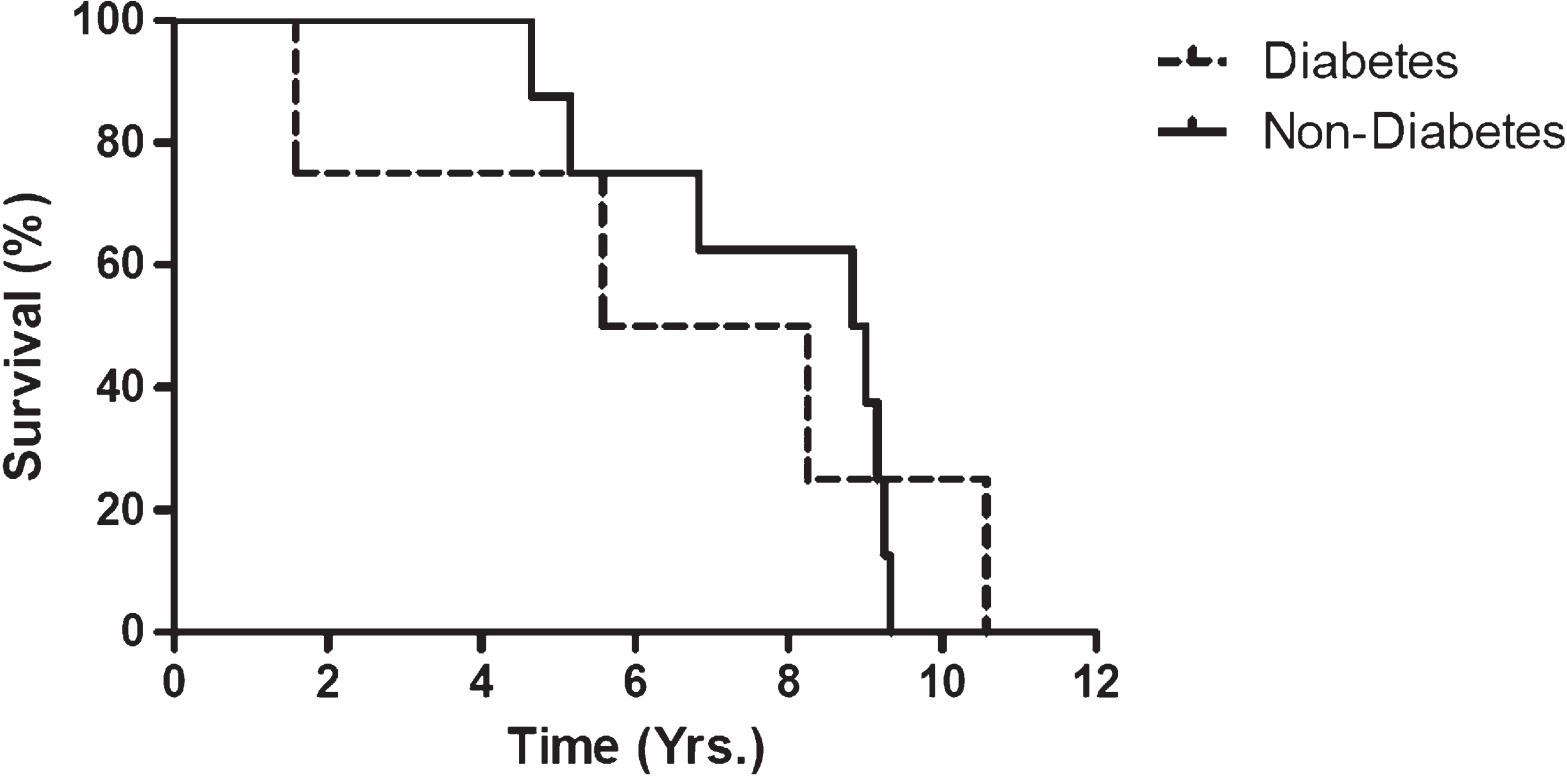
Kaplan Meier curve of all deaths in patients with diabetes vs. patients without diabetes.

A total of 12 of the original 60 patients (20%) died after the primary neurologic examination. Death occurred between 1 and 10 years after initial WNV infection. Of the deceased patients, all but one patient (92%) had WNE (Figure 2), and of the original 35 patients with WNE, 11 (31%) patients died. Ten of the patients who died had hypertension (83%) and 4 had diabetes mellitus (33%) (Figure 3

).

### Factors associated with an abnormal neurological exam.

At the time of the primary assessment, abnormal neurological exams (excluding hearing loss) were found in 2/10 (20%) WNF cases, 3/12 (25%) WNM cases, and 30/35 (86%) WNE cases. On univariate logistic regression analysis, an abnormal neurologic exam findings was significantly associated with having a history of WNE (Odds Ratio [OR] = 24.0, 95% confidence interval [CI] = 6.1–93.8; *P* < 0.0001) and with age 60 years and older (OR = 5.4, 95% CI = 1.7–16.9; *P* = 0.004). On multivariate logistic regression analysis, having a history of WNE was the only variable found to be independently associated with abnormal neurological exam findings. Comorbid conditions (hypertension, stroke, or diabetes) were not found to be statistically associated with an abnormal neurological exam. When examining differences in specific neurological findings between WNE and non-WNE cases, WNE cases were significantly more likely to have abnormal tandem gait compared with those who did not have encephalitis (OR = 1.9; 95% CI = 2.0–37.1; Fisher Exact *P* value = 0.001).

## Discussion

We found that patients who initially presented with acute WNE have high rates of persistent neurological abnormalities over time compared with those who presented with acute WNF or WNM. Even when controlling for age, having a clinical presentation of WNE remained statistically associated with having abnormal neurological exam findings. Some of the persistent abnormalities were found to be severe, including motor strength deficits, abnormal reflexes, hearing loss, and tandem gait abnormalities. Interestingly, 4/7 (57%) WNF, 2/6 (33%) WNM, and 5/14 (36%) WNE had new neurological abnormalities evident at the time of the second assessment. This is the largest report of neurological assessments among patients with clinical WNV infection and the first to systematically examine patients at 1–3 and 8–11 years after their acute infection.

Other studies have also evaluated the neurological outcomes in patients with WNND. Sejvar and others prospectively followed 16 patients with severe WNV infections. At 8 months postinfection, the five meningitis patients were fully recovered, and among the eight encephalitis patients, five recovered, two did not recover to preinfection neurologic status but survived, and one remained comatose and ventilator dependant.^10^ In a second study, Klee and others evaluated the recovery of patients affected by the 1999 outbreak of WNV in New York City through telephone surveys of patients at 12 months postinfection. By 12 months postinfection, only 37% had fully recovered, 49% had difficulty walking, and 44% had muscle weakness. Patients with a history of meningitis or milder infections had fewer long-term neurologic sequelae as compared with those patients with encephalitis,^14^ similar to the findings in our study.

Our study demonstrated that older age and a diagnosis of WNE were associated with persistent neurological abnormalities, which is consistent with the previous literature.^14^–^16^ Furthermore, our data showed that patients who died prior to the secondary neurologic assessment were more likely to have a history of WNE and were more likely to have comorbidities including hypertension. In a study by Klee and others, at 12 months follow-up from initial infection, age was a positive predictor of recovery, with younger persons more likely to achieve physical, cognitive, and functional recovery.^14^ Murray and others also described advanced age, male gender, and history of hypertension as significant risk factors for developing WNE and for death.^16^ Although the exact mechanisms for the association between age and comorbidities and severity of neuroinvasive disease is currently unknown, the data represent a reduced neurologic recovery potential in older patients and/or patients with comorbidities infected with WNV infection when compared with younger, healthier patients.

Of our WNE patients, approximately 37% at primary assessment and 21% at secondary assessment had evidence of neuromuscular weakness. Other studies have consistently found muscle weakness to be a common complication of WNND infections. In one study, motor deficits were found in 4 (50%) of 8 patients with WNE patients during acute infection.^10^,^15^ Sejvar and others found that muscle weakness persisted for at least 8 months in patients with WNND presenting as poliomyelitis-like syndrome. Follow-up electromyography (EMG) revealed chronic denervation and motor axon loss in these patients.^10^ In a recent study from Greece, Anastasiadou and others showed that 73% of the 22 patients with WNND had muscle weakness 16 months after the onset of symptoms.^17^

Abnormal deep tendon reflexes were also prevalent in our WNE patients at both the 1–3 and the 8–11 years follow-up. WNV has been reported to cause acute flaccid paralysis or WNV poliomyelitis-like syndrome, which is described as areflexia and focal flaccid weakness.^10^ Although decreased reflexes have been reported in studies evaluating WNND, these patients have also been diagnosed with acute flaccid paralysis. No patients in this cohort had symptoms consistent with WNV poliomyelitis-like syndrome; however several patients had isolated decreased reflexes. Published data regarding isolated decreased reflexes in WNE are sparse.

Poor balance was found to be the most common and persistent problem in patients with a history of WNV. Overall, 60% with WNE, 14% with WNM, and 18% with WNF had impaired tandem gait at 1–3 years follow-up. In the majority of patients, these findings persisted at 8–11 years follow-up. The etiology of this deficit is unknown and possibly multifactorial including deficits in motor function, neurosensory function, and vestibular function. Interestingly, out of the 60 patients only two patients had abnormal heel-to-shin testing and no patients had abnormal finger-to-nose or rapid alternating movements indicating all patients had intact coordination.

Several of our patients had tremors, including postural and intention (action) tremors. Sejvar and others reported Parkinson-like action tremors at the 8-month follow-up of WNE patients.^10^ In our Houston cohort, there were no patients with obvious signs of parkinsonism such as cogwheeling, resting tremor, or festinating gait. This is an interesting inconsistency between the two studies. The discrepancy may be related to the follow-up time interval as patients in the Houston cohort had their first evaluation at a later date after acute infection. This discrepancy in neurologic exam findings may suggest that Parkinson-like symptoms resolve with time.

Sensory deficits were also observed in high frequency in this study. However, given the high number of patients with comorbidities, particularly diabetes, the data are difficult to interpret in this study population. Similarly, while hearing loss was found in a number of our patients, it is impossible to determine if these auditory defects were due to WNV injury alone or secondary to previous hearing loss or age-related hearing loss. Further studies are needed to determine the impact of invasive WNV on vestibular function.

We did not observe any cases of viral relapse as has been described in other encephalitis. Herpes simplex encephalitis, for example, has been shown to have a relapse rate of approximately 10%.^18^ As a result of decreased potential for relapse and evidence of persistent neurologic deficits at 1–3 and 8–11 years postinfection documented in our study, we feel that recovery from WNV occurs mostly in the first year after infection with minimal recovery thereafter. It may be helpful to advise the WNE patients that the neurological deficits that persist at 1 year and beyond may not dissipate.

There are many limitations to our study. First, many of our findings could be attributable to the small number of patients when we examine subgroups. Because of the follow-up nature of the study, we had two different clinicians performing the study potentially resulting in interobserver reliability. This is thought be minimal, however, considering both clinicians received training on the proper examination techniques. Another inherent limitation is that the participants did not have a baseline neurological examination prior to their WNV infection, since infection could obviously not be predicted. Although the patient and family members were able to provide us with historical information about the patients' neurological status before their WNV infection, inaccuracies are possible due to recall bias. In addition, we are unable to delineate whether some of these abnormal neurological findings could be secondary to other causes such as diabetic neuropathy, stroke, or due to aging-related sensorineural hearing loss. Conducting a case–control study using age-matched normal controls could help establish a baseline group for comparison, but this could be cost-prohibitive and challenging. Larger scale multicenter studies would be of great benefit but may be impossible due to the waning of the epidemic. Finally, it is possible that a subset of WNF cases were misclassified because they did not have a lumbar puncture procedure at the time of acute illness, and therefore did not have a CSF analysis or brain imaging to determine if there was any evidence of meningitis or encephalitis.

In conclusion, we found that WNE, compared with WNF and WNM, is commonly associated with an abnormal neurological examination at both 1–3 and 8–11 years post-WNV infection, and we found that regardless of initial clinical presentation following WNV infection, new neurological abnormalities might develop over time. These abnormalities are chiefly abnormal reflexes, muscle weakness, gait impairment, and hearing and limb sensory loss. At present, there are no clear lines of prevention and treatment of WNV infection outside avoidance of mosquitoes and use of repellants. Through this observational study, the cohort has highlighted what may be unique neurologic characteristics associated with neuroinvasive West Nile virus disease and should prompt further assessment through prospective case–control studies. Our data will nevertheless be helpful to clinicians and patients to provide a prediction of long-term neurological complications following WNV infection, allowing for active case management to be planned and instituted.

## Supplementary Material

Supplemental Tables.

## Figures and Tables

**Table 1:** Demographic characteristics and prevalence of comorbid conditions in the Houston West Nile virus cohort at the time of acute West Nile infection as assessed during the primary neurological assessment

Demographic and comorbidity variables	WNF (*N* = 11)	WNM (*N* = 14)	WNE (*N* = 35)
Median age (range) in years	54 (17–76)	46 (22–65)	68 (9–86)
Male gender (%)	3 (27%)	9 (64%)	26 (74%)
Race/ethnicity (%)			
Caucasian	10 (91%)	12 (86%)	29 (83%)
African American	1 (9%)	2 (14%)	2 (6%)
Hispanic	0	0	4 (11%)
Diabetes mellitus	1 (9%)	2 (14%)	11 (31%)
History of stroke prior to WNV	0	1 (7%)	8 (22%)
Chronic alcohol use (> 15 drinks/week)	0	2 (14%)	4 (11%)
Hypertension	2 (14%)	3 (21%)	23 (65%)

WNF = West Nile fever; WNE = West Nile encephalitis; WNM = West Nile meningitis.

**Table 2:** Neurological examination results at primary follow up (1–3 years) post–West Nile virus infection: Prevalence of abnormal findings among the Houston West Nile cohort

Abnormal neurological exam findings	WNF (*N* = 11)	WNM (*N* = 14)	WNE (*N* = 35)	Total (*N* = 60)
Abnormal level of consciousness	0	0	0	0
Motor weakness	1 (9%)	0	13† (37%)	14† (23%)
Reflexes				
Tendon reflexes	0	1 (7%)	9† (26%)	10 (17%)
Babinski	0	0	1 (3%)	1 (2%)
Clonus	0		1 (3%)	1 (2%)
Sensory				
Light touch	0	1 (7%)	1 (3%)	1 (2%)
Pinprick	0	1 (7%)	4 (11%)	5 (8%)
Vibratory	0	2 (14%)	11 (31%)	13 (22%)
Cerebellum				
Heel/shin	0	0	2 (6%)	2 (3%)
Finger to nose	0	0	0	0
Rapid alternating movements	0	0	0	0
Tremor	0	1 (7%)	7 (20%)	8 (13%)
Romberg	1 (7%)	0	6 (17%)	7 (12%)
Gait	0	0	4 (11%)	4 (7%)
Tandem	2 (18%)	2 (14%)	21 (60%)	25 (42%)
Toe walking	1 (7%)	1 (7%)	7 (20%)	9 (15%)
Heel walking	1 (7%)	0	8 (23%)	9 (28%)
Cranial nerves				
Visual acuity	0	0	1 (3%)	1 (2%)
Facial asymmetry	0	0	0	0
Pupil reactivity	0	0	1* (3%)	1* (2%)
Hearing	2* (18%)	2* (14%)	16† (46%)	20† (33%)
Extraocular muscles	0	1* (7%)	1 (3%)	2† (3%)
Palate elevation	0	0	0	0
Facial sensation	0	1 (7%)	0	1 (2%)
Trapezius/sternocleidomastoid	0	0	1 (3%)	1 (2%)
Jaw strength	0	0	0	0
Hypoglossal/tongue	0	0	1 (3%)	1 (2%)

WNF = West Nile fever; WNE = West Nile encephalitis; WNM = West Nile meningitis.

* Abnormality prior to the WNV infection in all patients in cell.

† Abnormality prior to the WNV infection in some patients in the cell.
