# Supplementary material for: Long-Term Neurological Outcomes in West Nile Virus–Infected Patients: An Observational Study
Source: Am J Trop Med Hyg. 2015 May 6;92(5):1006–12. doi: 10.4269/ajtmh.14-0616 (PMC4426557; doi:10.4269/ajtmh.14-0616)
Supplement: Supplementary file 1 [file SD8.pdf]

Supplemental Table 1

Primary neurological exam findings on patients who had died prior to the time of the second assessment

Acute disease	Demographics	Comorbid conditions	Primary exam: Years Postinfection	Primary exam: Abnormal neurological findings
WNM	65-year-old male, Caucasian	Hypertension	3.8	Tremor in left hand
WNE	78-year-old male, Caucasian	Hypertension<br>Diabetes	2.2	Abnormal hearing on left ear<br>Abnormal pinprick and vibratory sensation<br>Abnormal Romberg<br>Symmetric decreased reflexes<br>Abnormal tandem gait
WNE	86-year-old female, Caucasian	Hypertension<br>Stroke	3	Abnormal hearing<br>Abnormal vibratory sensation<br>Abnormal Romberg<br>Symmetric, decreased reflexes
WNE	74-year-old male, Caucasian	Hypertension<br>Stroke	3.1	Hearing loss prior to WNV<br>Right leg weakness<br>Abnormal tandem gait
WNE	74-year-old male, Caucasian	Hypertension<br>Diabetes	3.1	Hearing loss prior to WNV<br>Bilateral 5th digit contracture<br>Abnormal Romberg<br>Symmetric decreased reflexes<br>Abnormal heel walk<br>Abnormal toe walk<br>Wide-based gait<br>Abnormal tandem gait
WNE	45-year-old male, Caucasian	Hypertension<br>ETOH	3.5	Symmetric increased reflexes<br>Tremor<br>Clonus present<br>Abnormal gait<br>Abnormal heel walk<br>Abnormal toe walk<br>Abnormal tandem gait
WNE	70-year-old male, Caucasian	Hypertension<br>Stroke<br>Diabetes	3.4	Hearing loss prior to WNV<br>Abnormal pinprick and vibratory sensation<br>Abnormal Romberg<br>Abnormal gait<br>Abnormal heel walk<br>Abnormal toe walk<br>Abnormal tandem gait<br>Abnormal heel to shin
WNE	73-year-old male, Caucasian	Hypertension<br>Stroke	2.8	Left arm weakness<br>Abnormal tandem gait
WNE	77-year-old male, Caucasian	None	1.8	Right pupil nonreactive since birth<br>Left ankle weakness<br>Tremor<br>Abnormal Romberg<br>Abnormal gait<br>Abnormal heel walk<br>Abnormal toe walk<br>Abnormal tandem gait<br>Abnormal heel to shin
WNE	55-year-old female, African American	Hypertension<br>Diabetes<br>Cataracts	1.1	Lethargic<br>Abnormal vision<br>Right leg weakness<br>Tremor<br>Abnormal vibratory sensation<br>Abnormal gait<br>Abnormal heel walk<br>Abnormal toe walk<br>Abnormal tandem gait
WNE	79-year-old male, Caucasian	None	3.4	Hearing loss in left ear<br>Abnormal light touch, pinprick, and vibratory sensation<br>Abnormal tandem gait
WNE	68-year-old male, Caucasian	Hypertension<br>Stroke	0.8	Bilateral nystagmus<br>Decreased reflexes on left Achilles, increased symmetric reflexes biceps<br>Abnormal tandem gait

ETOH = chronic alcohol use; WNF = West Nile fever; WNE = West Nile encephalitis; WNM = West Nile meningitis.

Supplemental Table 2

Neurological abnormalities observed during both primary and secondary assessments among participants in the Houston West Nile Virus cohort

Acute disease	Demographics	Comorbid conditions	First exam: Years postinfection	First exam: Abnormal neurological findings	Second exam: Years Postinfection	Second exam: Abnormal neurological findings
WNF	75-year-old female, Caucasian	None	3.0	Abnormal hearing, unknown date of onset<br>Abnormal tandem gait	11.2	Nonambulatory, nonverbal, aphasic, unresponsive to commands, severe contractions of all extremities with increased tone
WNF	52-year-old male, Caucasian	None	1.1	Normal exam	8.3	Abnormal pinprick sensation<br>Left finger weakness<br>Abnormal tandem gait
WNF	17-year-old female, Caucasian	None	1.9	Normal exam	9.3	Abnormal pinprick sensation
WNF	50-year-old female, Caucasian	Hepatitis B	0.9	Right arm weakness<br>Abnormal Romberg<br>Abnormal heel walk<br>Abnormal toe walk<br>Abnormal tandem gait	8.3	Abnormal pinprick sensation<br>Abnormal Romberg<br>Abnormal heel walk<br>Abnormal toe walk<br>Abnormal tandem gait
WNM	58-year-old female, African American	Hypertension<br>Diabetes	3.3	Abnormal sensation to pinprick<br>Abnormal vibratory sensation<br>Abnormal tandem gait	9.8	Abnormal sensation to pinprick<br>Abnormal vibratory sensation<br>Abnormal tandem gait
WNM	48-year-old male, Caucasian	ETOH<br>Hepatitis C	3.1	Abnormal hearing prior to WNV<br>Abnormal tandem gait	10.2	Abnormal hearing<br>Abnormal pinprick sensation bilateral lower extremities<br>Bilateral lower extremity weakness<br>Abnormal tandem gait
WNM	49-year-old male, Caucasian	None	2.3	Normal exam	8.9	Abnormal pinprick sensation bilateral lower extremities
WNE	78-year-old male, Caucasian	Hypertension	0.8	Abnormal hearing prior to WNV<br>Abnormal tandem gait	8.0	Abnormal hearing<br>Symmetric decreased reflexes in upper and lower extremities<br>Abnormal tandem gait
WNE	76-year-old female, Caucasian	Hypertension<br>Diabetes	3.1	Abnormal hearing prior to WNV<br>Abnormal tandem gait	10.4	Abnormal hearing prior to WNV<br>Abnormal tandem gait
WNE	60-year-old male, Caucasian	Hypertension<br>CVA<br>ETOH	3.0	Symmetric decreased reflexes in lower extremities	10.4	Symmetric decreased reflexes in upper extremities and decreased reflexes in left lower extremity
WNE	64-year-old male, Caucasian	Hypertension<br>ETOH	2.9	Abnormal hearing prior to WNV<br>Fine tremor	10.8	Abnormal hearing prior to WNV<br>Fine tremor
WNE	49-year-old male, Caucasian	None	1.8	Left upper extremity weakness<br>Symmetric decreased reflexes	9.3	Left upper extremity weakness<br>Symmetric decreased reflexes<br>Left upper extremity tremor
WNE	68-year-old male, Caucasian	Hypertension	3.2	Abnormal hearing<br>Abnormal tandem gait<br>Fine tremor	10.9	Abnormal hearing<br>Symmetric decreased strength<br>Tremor resolved<br>Abnormal tandem gait
WNE	70-year-old male, Caucasian	Hypertension<br>CVA	3.1	Left ankle weakness<br>Decreased right quadriceps and increased left quadriceps reflexes<br>Abnormal Babinski<br>Abnormal Romberg<br>Abnormal heel walk<br>Abnormal tandem gait	10.5	Decreased right quadriceps and increased left quadriceps reflexes<br>Abnormal Babinski<br>Abnormal Romberg<br>Abnormal heel walk<br>Abnormal tandem gait
WNE	51-year-old male, Hispanic	Diabetes	2.9	Decreased reflexes on right upper extremity, bilateral lower extremities<br>Abnormal tandem gait	10.3	Symmetric decreased upper extremity reflexes<br>Abnormal tandem gait
WNE	72-year-old male, Caucasian	Hypertension<br>Diabetes	2.8	Tongue deviation to right<br>Abnormal hearing<br>Abnormal vibratory sensation<br>Abnormal heel walk<br>Abnormal toe walk<br>Abnormal tandem gait	10.6	Tongue deviation resolved<br>Abnormal hearing<br>Abnormal heel walk<br>Abnormal toe walk<br>Abnormal tandem gait
WNE	67-year-old female, Caucasian	CVA	2.3	Right upper extremity weakness	9.0	Right upper extremity and left hand weakness<br>Decreased right bicep, brachioradialis, tricep reflexes, and decreased left tricep reflex

ETOH = chronic alcohol use; WNF = West Nile fever; WNE = West Nile encephalitis; WNM = West Nile meningitis.
